# Phytotoxic Effects of Polystyrene and Polymethyl Methacrylate Microplastics on *Allium cepa* Roots

**DOI:** 10.3390/plants12040747

**Published:** 2023-02-07

**Authors:** Renata Biba, Petra Cvjetko, Mihaela Jakopčić, Bruno Komazec, Mirta Tkalec, Nino Dimitrov, Tajana Begović, Biljana Balen

**Affiliations:** 1 Department of Biology, Faculty of Science, University of Zagreb, Horvatovac 102a, 10000 Zagreb, Croatia; 2 Croatian Institute for Public Health, Rockefellerova 7, 10000 Zagreb, Croatia; 3 Department of Chemistry, Faculty of Science, University of Zagreb, Horvatovac 102a, 10000 Zagreb, Croatia

**Keywords:** microplastics, reactive oxygen species, oxidative stress, antioxidant enzymes, onion

## Abstract

Plastic contamination has become one of the most pressing environmental issues due to rapidly increasing production of disposable plastic products, their fragmentation into smaller pieces, and long persistence in the environment, which affects all living organisms, including plants. In this study, *Allium cepa* roots were exposed to 0.01, 0.1, and 1 g L^−1^ of commercial polystyrene (PS-MPs) and polymethyl methacrylate microparticles (PMMA-MPs) for 72 h. Dynamic light scattering (DLS) analyses showed high stability of both types of MPs in ultrapure water used for *A. cepa* treatment. Morphometric analysis revealed no significant change in root length compared to control. Pyrolysis hyphenated to gas chromatography and mass spectrometry (Py-GC-MS) has proven PS-MPs uptake by onion roots in all treatments, while PMMA-MPs were recorded only upon exposure to the highest concentration. Neither MPs induced any (cyto)toxic effect on root growth and PMMA-MPs even had a stimulating effect on root growth. ROS production as well as lipid and protein oxidation were somewhat higher in PS-MP treatments compared to the corresponding concentrations of PMMA-MP, while neither of the applied MPs induced significant damage to the DNA molecule assayed with a Comet test. Significantly elevated activity of H_2_O_2_ scavenging enzymes, catalase, and peroxidases was measured after exposure to both types of MPs. Obtained results suggest that onion roots take up PS-MPs more readily in comparison to PMMA-MPs, while both types of MPs induce a successful activation of antioxidant machinery in root cells that prevented the occurrence of toxic effects.

## 1. Introduction

Plastic demand is continuously growing in different industrial areas such as construction, packaging, automotive, agriculture, mechanical engineering, medical applications, electronics, etc. Data from 2020 indicate that 367 million tons of different plastic materials were produced in the world during that year (https://plasticseurope.org/ accessed on 21 December 2022), with a large proportion of these ending up in the environment as waste, thus generating a relevant environmental challenge. Moreover, during the COVID-19 pandemic, the increased production of disposable protective equipment resulted in an annual generation of 75 kg of plastic waste per capita [1,2]. Since plastics are not biodegradable, they remain in the environment for a very long time and, exposed to environmental stress (mechanical stress, temperature, and UV exposure), undergo fragmentation into smaller pieces, from larger plastic debris down to micro (from 100 nm to 5 mm, MPs) and nano (less than 100 nm, NPs) particle dimension [3,4]. Recently, a more precise categorization has been suggested: micro (5 mm–1 μm), submicron (1 μm–100 nm), and nanoplastic (<100 nm) [5]. Particulate plastics persist in terrestrial (i.e., soil) and aquatic (i.e., marine and freshwater) environments as well as in the atmospheric environment (i.e., atmospheric fallout) [6], endangering the whole living world. Plastic dimensions as well as the basic polymer structure are crucial variables in determining the level of toxicity of plastics in exposed organisms [7]. The most commonly identified MPs in the environment originate from polystyrene (PS), polyethylene terephthalate (PET), polyethylene (PE), polypropylene (PP), polyvinyl chloride (PVC), polymethyl methacrylate (PMMA), polyester (PES), and polyvinyl butyral (PVB) [8,9]. MP accumulation in different tissues of living organisms or drinking water has been reported to have a considerable impact on the food web [10,11]. The biological response to MP exposure has been studied in different models, and in some cases, serious toxic effects related to MP uptake were found in marine [12,13,14,15] and freshwater organisms [16], as well as in humans [17], although some studies reported low or non-existent toxic effects of MPs [18,19].

Numerous agricultural plants as well as grasses and aquatic plants were shown to be highly vulnerable to MPs [20]. It was found that MPs were stacked and adsorbed onto the root surface, but also entered the shoots of plants through the root system along with water and nutrients, using the transpiration pull of the vascular system [21,22,23]. Therefore, MPs might accumulate in the edible and non-edible parts of the plants [24]. Studies have shown that the phytotoxic effects of MPs on terrestrial plants are highly influenced by their physico-chemical characteristics, primarily the polymer type [21]. The majority of the studies were performed using PS-MPs, which mostly resulted in a negative impact. Namely, reduced germination, plant growth, and biomass were recorded in *Lepidium sativum* [21], while induced cytotoxicity, genotoxicity, and oxidative damage were found in *Vicia faba* [22], *Lactuca sativa* [25], and *Allium cepa* [26]. Studies performed with other types of MPs also exhibited a detrimental impact on plants. PMMA-MPs significantly inhibited the germination index of *Brassica campestris* [23]. Exposure to PE-MPs resulted in a damaged antioxidant system in *Triticum aestivum* roots [27], reduced or blocked water and nutrient uptake as well as the growth of the *Zea mays* plants, [28] and increased toxicity, uptake, accumulation, and bioavailability of heavy metals in lettuce [29]. Moreover, Teng et al. [30] reported harmful effects of PE-MPs on the photosynthetic performance in leaves of *Nicotiana tabacum* seedlings.

However, plant exposure to MPs might not always exhibit negative effects. Boots et al. [31] observed no changes in germination rate and chlorophyll content in *Lolium perenne* treated with high-density PE-MPs, while the 48 or 72 h exposure of *L. sativum* to NPs and MPs failed to reveal significant differences in root growth [21]. The lack of effect on root growth was also reported for *A. cepa* [32], *V. faba* [22], and *Cucumis sativus* [33] upon exposure to PS-MPs. Moreover, in some studies, even positive, acclimative responses were recorded. Namely, results obtained on *Allium fistulosum* [34] and *T. aestivum* [35] upon exposure to PS-MPs as well as on *L. perene* treated with PE-MPs [31] showed increased root length and biomass. Furthermore, exposure of *L. sativa* to PVC-MPs significantly increased total root length, surface area, volume, and diameter as well as SOD activity [25]. These findings indicate that MP-induced effects might have variable outcomes depending on the plant species. The reasons for variable plant responses are not completely elucidated and it is, therefore, difficult to predict the global impact of MPs and NPs on primary producer species [36], which justifies further research on MPs’ potential phytotoxic effects.

In the present study, we conducted a parallel plant exposure to MPs composed of two different polymers, polystyrene (PS) and polymethyl methacrylate (PMMA). Polystyrene is one of the leading pollutants found in both aquatic and terrestrial ecosystems [32], which is why the effects of PS-MPs and PS-NPs are often investigated in toxicological studies on plants. On the contrary, although PMMA is commonly used, for example for microbeads in cosmetics in Europe [37,38], only a few publications studying the effects of PMMA-MPs on plants can be found in the literature [38,39]. The aim of this research was to test the phytotoxicity of both types of MPs and to reveal if MPs originating from different polymers might induce similar or diverse effects in the same plant species. As the model organism, we chose onion (*Allium cepa*), one of the most important plants grown and consumed all over the world, but also a very common model in abiotic stress research [40,41,42]. To better examine the phytotoxicity induced by two types of MP in *A. cepa* roots, we applied MP concentrations determined by our preliminary experiments and data obtained from the literature [22,26,39,43], rather than those discerned in the natural environment.

## 2. Results

### 2.1. MP Characterization and Stability

**Figure 1:**
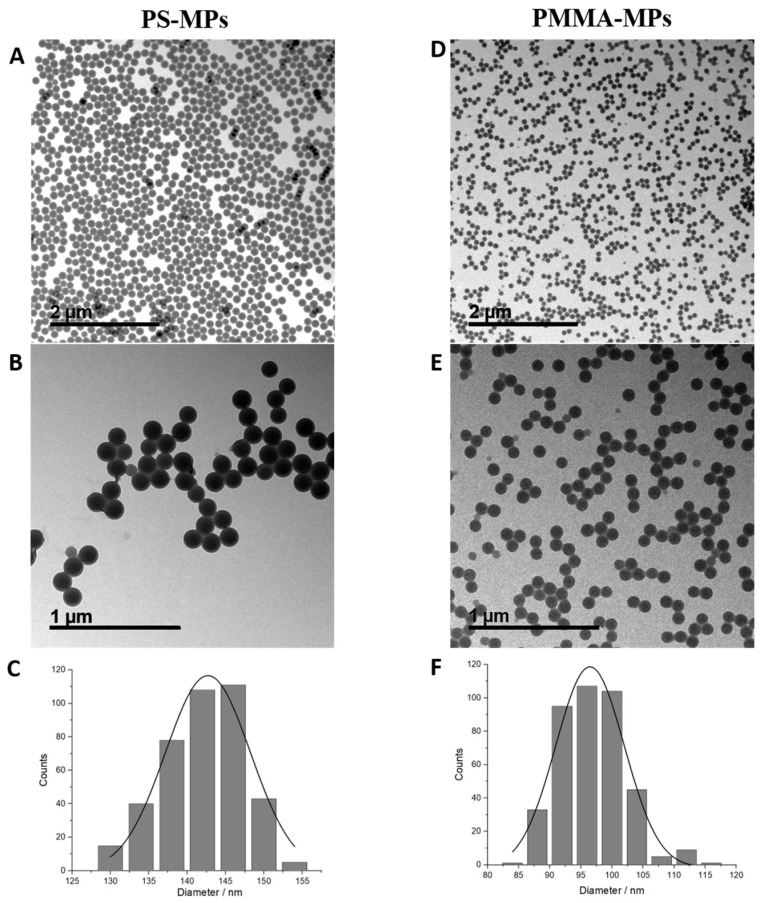
Stock solutions of polystyrene (PS) and polymethyl methacrylate (PMMA) microparticles in ultrapure water analyzed by transmission electron microscopy (TEM); bright field images (**A**,**B**,**D**,**E**); size distribution histograms (**C**,**F**). Three replicas (*n* = 3) were investigated for each stock solution. Scale bar represents 2 µm (**A**,**D**) or 1 µm (**B**,**E**).

Microphotographs obtained by transmission electron microscopy (TEM) imaging show that PS-MPs (Figure 1A,B) and PMMA-MPs (Figure 1D,E) are spherical in shape, while size distribution analysis indicates that both types of MPs are mostly uniform in diameter, with mean values of 142.70 ± 5.53 nm (PS-MPs, Figure 1C) and 96.46 ± 5.52 nm (PMMA-MPs, Figure 1F).

Temporal DLS analysis of 0.01, 0.1, and 1 g L^−1^ PS-MPs and PMMA-MPs revealed their behavior in ultrapure water (Table 1), which was used for the exposure treatments of onion roots. At the lowest PS-MP concentration, their size increased after the first 24 h compared to 0 min, and then remained stable, while at 0.1 g L^−1^ d_H_, the value first increased after 24 h and then decreased. The higher PS-MP concentration exhibited a gradual decrease in d_H_ values. As for the PMMA-MPs, at the 0.01 g L^−1^ concentration, MP size increased at 48 h and then decreased at 72 h compared to 0 min. Analyses of two higher PMMA-MP concentrations revealed a reduction in d_H_ values in comparison to 0 min.

### 2.2. Root Growth, Mitotic Index, and Chromosomal Aberrations

**Figure 2:**
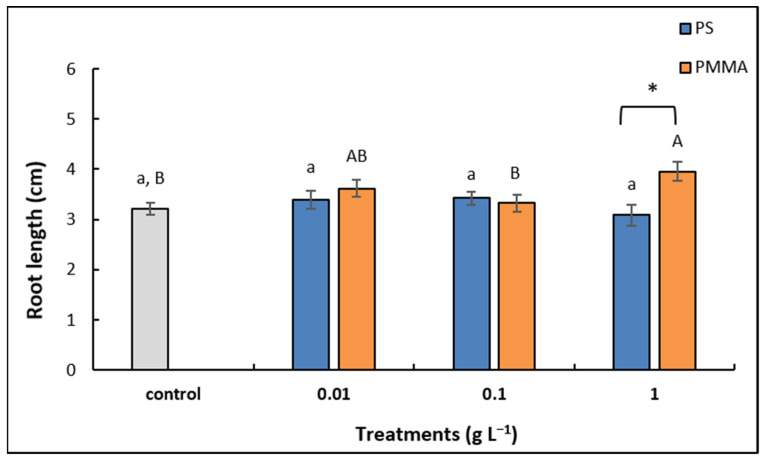
Length of *A. cepa* control roots and after 72 h treatment with 0.01, 0.1, and 1 g L^−1^ of PS-MPs and PMMA-MPs. Values are means ± standard errors of two different experiments, each with 6 replicas (*n* = 12). If columns are marked with different letters or asterisk, the treatments are significantly different at *p* ≤ 0.05 (one-way ANOVA followed by Duncan post hoc test); small letters mark the differences among different concentrations of PS-MPs and control, capital letters mark the differences among different concentrations of PMMA-MPs and control, and asterisk (*) denotes difference between the same concentration of different MP types.

Morphometric analysis revealed no significant changes in root growth upon exposure to any of the investigated PS-MP concentrations compared to control, although the highest concentration (1 g L^−1^) induced a slight decrease in length. Interestingly, among the treatments with PMMA-MPs, the highest concentration (1 g L^−1^) resulted in significantly enhanced root growth in comparison to control. When treatments with corresponding concentrations of different MPs were compared, a significant increase in root length was recorded upon exposure to 1 g L^−1^ PMMA-MPs compared to PS-MPs (Figure 2).

Mitotic index was not significantly affected by any of the treatments with either PS-MPs or PMMA-MPs in comparison to control (Table 2). An increased number of cells with chromosomal abnormalities (in % of total dividing cells) was observed in all treatments, although exposure to the 1 g L^−1^ of both types of MPs resulted significantly in the highest values. Among the different types of chromosomal abnormalities which were recorded, a significant effect was found only for C-metaphase (C-mitosis), which was increased in all treatments compared to control; significantly, the highest number of cells with C-mitosis was recorded after treatments with the highest concentrations of both MP types (Table 2).

### 2.3. MP Uptake by Root Cells

PS-MP and PMMA-MP uptake by *A. cepa* roots was analyzed by pyrolysis–gas chromatography–mass spectrometry (Py-GC-MS) in order to verify if root cells can accumulate microparticles based on certain types of plastic polymers.

**Figure 3:**
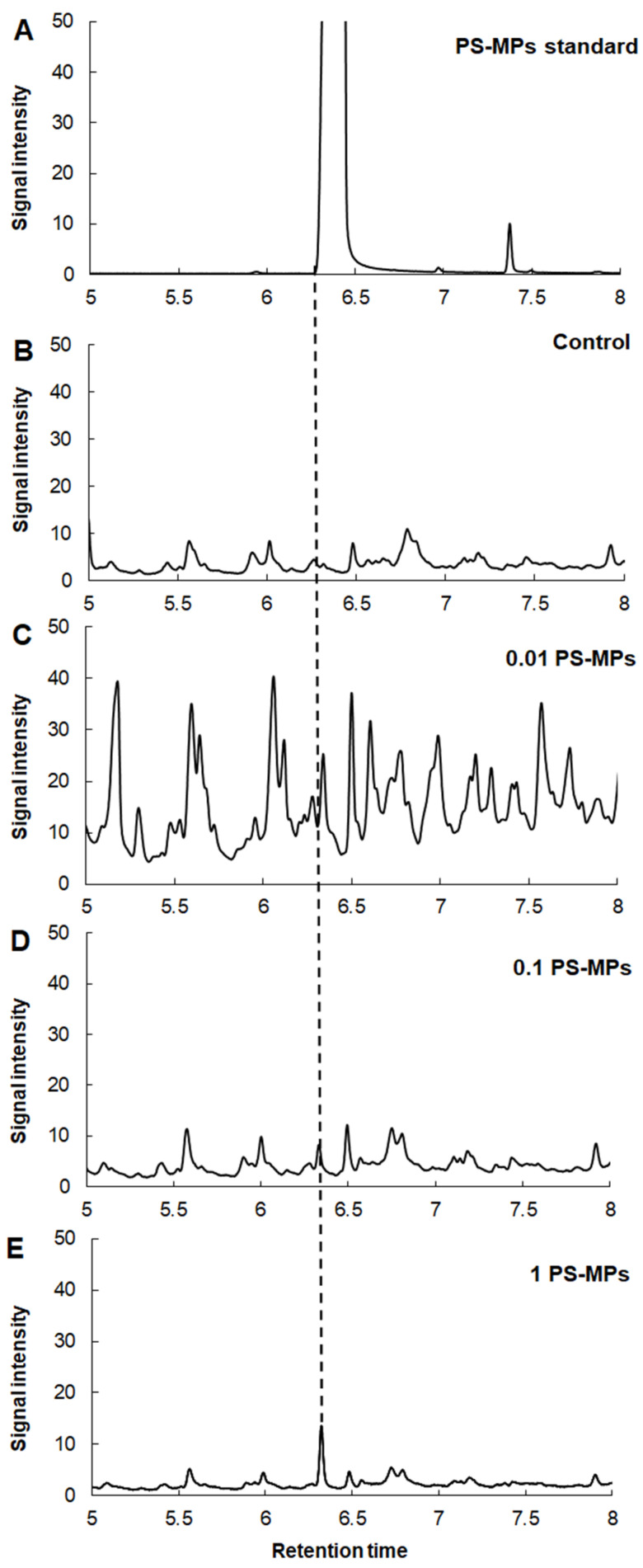
Extracted ion chromatogram of polystyrene monomer from pyrolysis GC-MS. Standard sample of PS-MPs (**A**); control *A. cepa* roots (**B**); *A. cepa* roots treated with 0.01 g L^−1^ PS-MPs (**C**), 0.1 g L^−1^ PS-MPs (**D**), and 1 g L^−1^ PS-MPs (**E**).

The main observed pyrolysis degradation products at 600 °C from the PS-MP standard were styrene monomer (M), dimer (D), and trimer (T) (Figure S1). A very prominent styrene monomer peak from the PS-MP standard (commercial monodisperse PS-MP solution used for exposure experiment) with characteristic ions *m/z* 51, 78, 104 was observed at 6.407 min (Figure 3A). A styrene dimer peak with characteristic ion *m/z* 91 was recorded at 13.487 min, while a very small peak intensity of styrene trimer (T) with characteristic ions *m/z* 91 was observed at 17.493 min (Figure S1). Moreover, styrene monomer peaks were observed in all analyzed root samples at 6.342, 6.333, and 6.327 min upon exposure to 0.01, 0.1, and 1 g L^−1^ of PS-MPs (Figure 3C,D), respectively. However, a small styrene monomer peak was also recorded in control *A. cepa* roots, which were not treated with PS-MPs (Figure 3B), although it was far less pronounced compared to standard and treatments.

**Figure 4:**
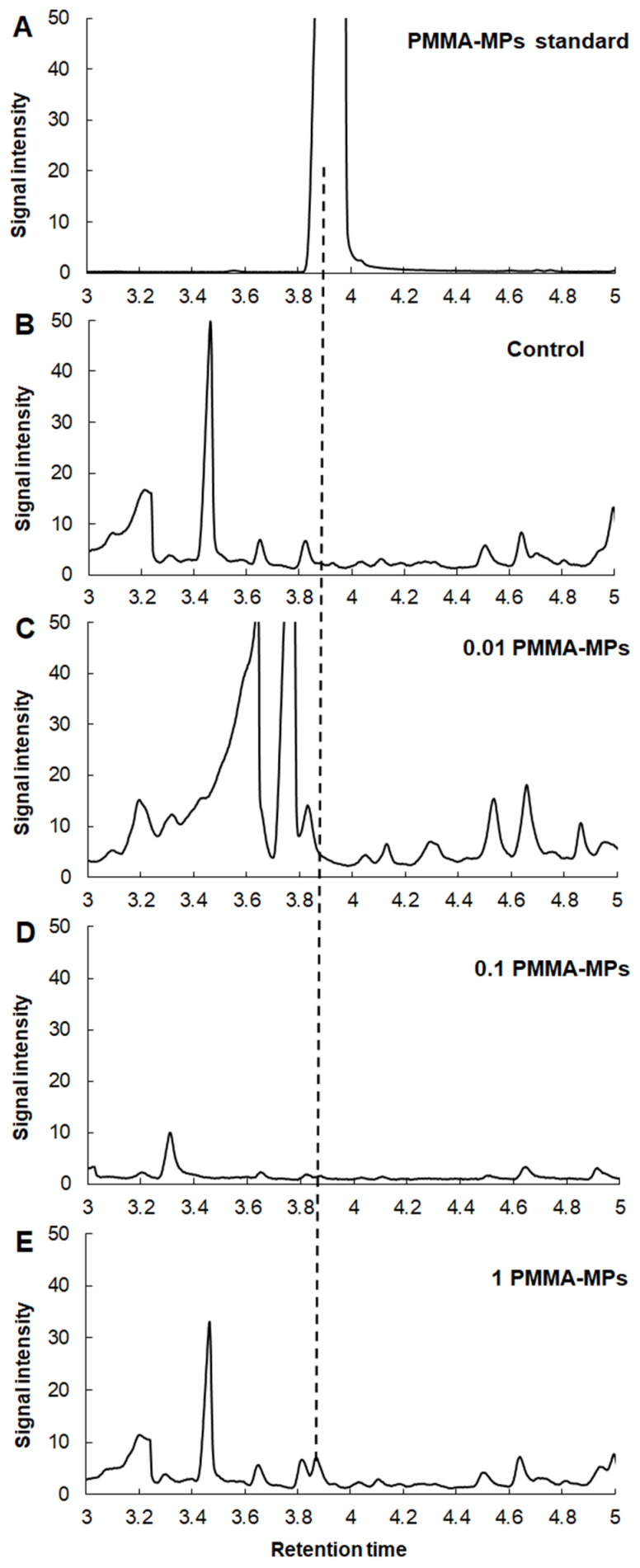
Extracted ion chromatogram of methyl methacrylate monomer from pyrolysis GC-MS. Standard sample of PMMA-MPs (**A**); control *A. cepa* roots (**B**); *A. cepa* roots treated with 0.01 g L^−1^ PMMA-MPs (**C**), 0.1 g L^−1^ PMMA-MPs (**D**), and 1 g L^−1^ PMMA-MPs (**E**).

The main observed pyrolysis degradation product at 600 °C from the PMMA-MP standard (commercial monodisperse PMMA-MP solution used for exposure experiment) was methyl methacrylate (Figure 4A). The thermal degradation products of PMMA-MP pyrolysis with its characteristic ions of *m/z* 41, 69, and 100 (Figure S2) were detected only in the standard sample (Figure 4A) and roots exposed to 1 g L^−1^ PMMA-MPs (Figure 4E) at 3.958 and 3.872 min, respectively.

### 2.4. ROS Content

**Figure 5:**
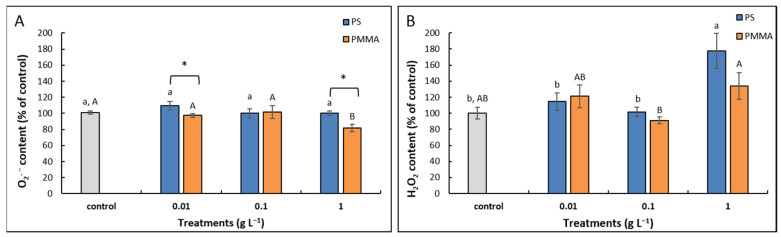
Content of O_2_^•−^ (**A**) and H_2_O_2_ (**B**) in *A. cepa* control roots and after 72 h treatment with 0.01, 0.1, and 1 g L^−1^ of PS-MPs and PMMA-MPs. Values are means ± standard errors of two different experiments, each with 6 replicas (*n* = 12). If columns are marked with different letters or asterisk, the treatments are significantly different at *p* ≤ 0.05 (one-way ANOVA followed by Duncan post hoc test); small letters mark the differences among different concentrations of PS-MPs and control, capital letters mark the differences among different concentrations of PMMA-MPs and control, and asterisk (*) denotes difference between the same concentration of different MP types.

Exposure to PS-MPs applied in three different concentrations failed to induce any significant change in the content of superoxide radical (O_2_^•−^) in comparison to control. Treatments with PMMA-MPs resulted in slightly decreased values at the lowest (0.01 g L^−1^) concentration and significantly decreased values at the highest tested concentration (1 g L^−1^). The comparison of corresponding treatments with different types of MPs revealed that roots treated with PS-MPs had higher O_2_^•−^ content than roots treated with PMMA-MPs at the lowest concentration and the highest concentration (Figure 5A).

As for the H_2_O_2_ content, somewhat different results compared to O_2_^•−^ content were obtained. Namely, treatment with 1 g L^−1^ PS-MPs resulted in a significantly elevated value when compared to control and other PS-MP treatments. Among treatments with PMMA-MPs, again, only the highest concentration induced the production of H_2_O_2_, which was not statistically significant in comparison to control, but was significantly higher compared to treatment with 0.1 g L^−1^ PMMA-MPs (Figure 5B).

### 2.5. Oxidative Damage of Lipids, Proteins, and DNA

**Figure 6:**
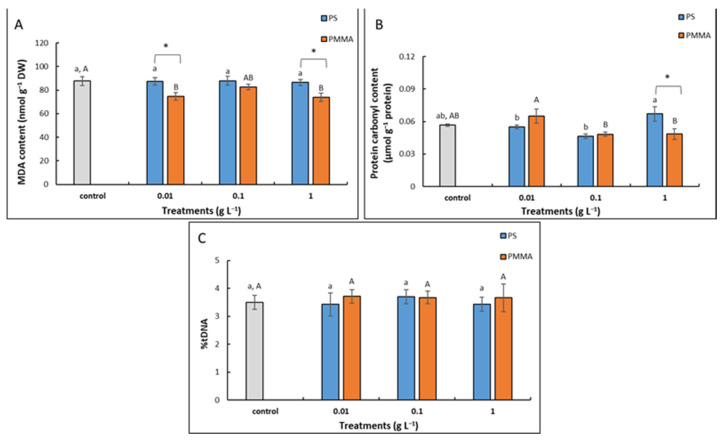
Content of malondialdehyde (MDA) (**A**), protein carbonyls (**B**), and %tDNA (**C**) in *A. cepa* control roots and after 72 h treatment with 0.01, 0.1, and 1 g L^−1^ of PS-MPs and PMMA-MPs. Values are means ± standard errors of two different experiments, each with 6 replicas (*n* = 12). If columns are marked with different letters or asterisk, the treatments are significantly different at *p* ≤ 0.05 (one-way ANOVA followed by Duncan post hoc test); small letters mark the differences among different concentrations of PS-MPs and control, capital letters mark the differences among different concentrations of PMMA-MPs and control, and asterisk (*) denotes difference between the same concentration of different MP types.

Treatments with PS-MPs resulted in MDA content values similar to control. On the contrary, all exposures to PMMA-MPs resulted in decreased values, although only at the lowest (0.01 g L^−1^) and the highest (1 g L^−1^) concentration were results statistically significant in comparison to control as well as to corresponding treatments with PS-MPs (Figure 6A).

Exposure to PS-MPs in any of the tested concentrations failed to show significant changes in protein carbonyl content in comparison to control; however, the values obtained upon treatment with the highest concentration (1 g L^−1^) were significantly elevated when compared to exposures with two lower concentrations. As for the treatments with PMMA-MPs, again, no significant changes in protein carbonyl content were recorded in comparison to control; however, the lowest concentration (0.01 g L^−1^) resulted in a significant increase compared to exposure to 0.1 and 1 g L^−1^ PMMA-MPs. The comparison of corresponding treatments with different types of MPs revealed a significantly higher value of protein carbonyls upon exposure to 1 g L^−1^ PS-MPs compared to PMMA-MPs (Figure 6B).

A Comet assay showed no significant changes in %tDNA upon treatments with PS-MPs or PMMA-MPs compared to control (Figure 6C).

### 2.6. Activities of Antioxidant Enzymes

**Figure 7:**
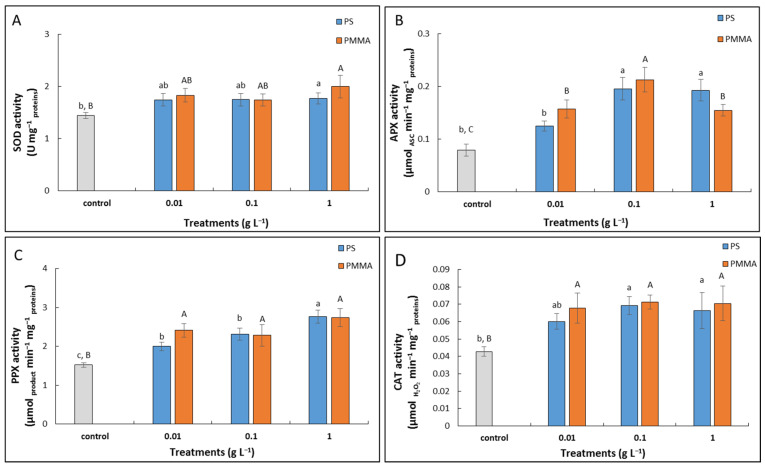
Activities of superoxide dismutase (SOD) (**A**), ascorbate peroxidase (APX) (**B**), pyrogallol peroxidase (PPX) (**C**), and catalase (CAT) (**D**) in *A. cepa* control roots and after 72 h treatment with 0.01, 0.1, and 1 g L^−1^ of PS-MPs and PMMA-MPs. Values are means ± standard errors of two different experiments, each with 6 replicas (*n* = 12). If columns are marked with different letters or asterisk, the treatments are significantly different at *p* ≤ 0.05 (one-way ANOVA followed by Duncan post hoc test); small letters mark the differences among different concentrations of PS-MPs and control, and capital letters mark the differences among different concentrations of PMMA-MPs and control.

Activity of superoxide dismutase (SOD; EC 1.15.1.1) revealed the same trend upon exposure to different types of MPs; values were elevated in all treatments with either PS-MPs or PMMA-MPs compared to control, although only the highest concentration (1 g L^−1^) of each MP type resulted in a significantly higher value (Figure 7A).

Treatments with PS-MPs resulted in elevated activity of ascorbate peroxidase (APX; EC 1.11.1.11) in comparison to control, although only higher concentrations (0.1 and 1 g L^−1^) revealed statistical significance. The increase in APX activity was even more pronounced upon exposure to PMMA-MPs, where all concentrations resulted in a significant increase when compared to control. Moreover, treatment with 0.1 g L^−1^ concentration induced the highest APX activity, which was significantly elevated compared to 0.01 and 1 g L^−1^ PMMA-MPs (Figure 7B).

Pyrogallol peroxidase (PPX; EC 1.11.1.7) activity was found to be significantly elevated upon exposure to all PS-MP concentrations in comparison to control; moreover, the highest concentration (1 g L^−1^) resulted in a value that was significantly elevated compared to treatments with the two lower PS-MPs concentrations. Treatments with PMMA-MPs also significantly induced PPX activity compared to the control, but to a similar extent at all tested concentrations (Figure 7C).

Exposure to PS-MPs revealed an increase in catalase (CAT; EC 1.11.1.6) activity at all tested concentrations compared to control, with significantly elevated values at two higher concentrations (0.1 and 1 g L^−1^). All treatments with PMMA-MPs significantly elevated CAT activity compared to the control to a similar extent at all tested concentrations (Figure 7D).

## 3. Discussion

### 3.1. MP Stability in Exposure Medium and Uptake

In this study, we used MPs based on two different polymers, PS-MPs and PMMA-MPs, whose sizes (147 and 105 nm, respectively) were at the limit between MPs and NPs. Temporal DLS analysis of both types of MPs revealed their good stability in ultrapure water applied for the exposure treatments of onion roots. This is in agreement with DLS analyses of differentially charged PS-NPs, when their optimal dispersion and stability in deionized water was confirmed [43]. However, in time we noticed some increase in size at the lowest applied concentration of both types of MPs and a decrease at two higher concentrations, which can be a consequence of the MPs aging [44]. Nevertheless, since in most of the DLS measurements MP sizes exceeded 100 nm, which is in agreement with the producer declaration, we classified them to MPs rather than to NPs according to the common size classification [3,4]. Although a more precise size classification of plastic particles into micro (5 mm–1 μm), submicron (1 μm–100 nm), and nanoplastics (<100 nm) has been recommended [45,46], the term “submicron” is still not widely accepted and the classification to micro and nanoplastics prevails, particularly in studies performed on plants.

Uptake in *A. cepa* roots of both types of MPs was analyzed by pyrolysis GC-MS, a technique which was already successfully applied to identify microplastics in some environmental samples, such as seawater and beach sediments [47] as well as in seafood [48] and polychaeta worms [49]. As for the plants, only a few studies can be found in which Py-GC-MS was applied to confirm MP uptake upon exposure. Taylor et al. [50] used Py-GC-MS to reveal PS-MP uptake in wheat and Arabidopsis, while Li et al. [51] employed the same technique for quantification of PS- and PMMA-MPs in cucumber plants.

In our study, a styrene monomer peak was observed in all treated samples, confirming the uptake of PS-MPs in *A. cepa* roots. Unexpectedly, the presence of a small styrene monomer peak was also observed in the control, but as it was very hard to distinguish it from the background, it can be assumed that the styrene peak signal detected in the treated *A. cepa* roots originates from the PS-MPs taken up by root cells. The formation of styrene as a pyrolysis degradation product of untreated plant material was also reported by other authors [50,52,53]. Styrene can be produced naturally in plants from the decarboxylation of cinnamic acid [54]. It has been shown that low levels of styrene may be found in fruits, cereals, and coffee beans as well as in olive oil [55] as a result of the biodegradation of naturally occurring compounds with structures similar to styrene [56]. Other than the styrene monomer, styrene trimer ion *m/z* 91 can also be used for the detection and quantification of PS-MPs [57]. However, we could not use it for MP detection because the PS-MP standard at 600 °C showed a very small peak intensity of styrene trimer (T) and because the characteristic ion for dimer (D) is also *m/z* 91 (Figure S1). Nevertheless, since the styrene monomer peak detected in the control root sample was very weak compared to samples of treated roots, we can conclude that it can be used as a proof of PS-MPs uptake by *A. cepa* root cells. Uptake of PS-MPs has been proven in other plants including rice [58], Arabidopsis [43], and onion [26].

Unlike polystyrene polymer, PMMA has been much easier to detect because it has no background interference. Other than the standard sample, characteristic ions of thermal degradation products were only recorded in roots exposed to 1 g L^−1^ PMMA-MPs, which might suggest that onion root cells take up PMMA-based microplastics less efficiently, and therefore, the Py-GC-MS method may not be sensitive enough to detect the methyl methacrylate signal in lower concentrations. Li et al. [51] also reported that the Py-GC-MS method was more sensitive for the detection and quantification of 50 nm PS-NPs compared to PMMA-MPs of the same size in roots of cucumber plants. The uptake of fluorescent-labelled PMMA-MPs by root cells was found in lettuce plants by scanning electron microscopy [59].

### 3.2. Effects on Root Growth

The root is an important plant organ responsible for water and nutrient uptake, but it is also essential for sensing the alterations in the external conditions, which allows the plant organism to quickly adapt to various environmental stresses [60]. In this study, no inhibitory effect on onion root growth was observed after exposure to 147 nm sized PS-MPs. This is in agreement with results obtained on *A. cepa* roots exposed to PS-MPs of sizes ranging from 80 to 8000 nm [32], as well as on *V. faba* [22] and *C. sativus* roots [33] treated with 100 nm sized PS-MPs. On the other hand, a positive effect on root elongation of wheat seedlings was reported upon exposure to 100 nm PS-MPs [35]. On the contrary, 100 nm PS-MPs significantly reduced *A. cepa* root length, which has been ascribed to MP-induced cytogenotoxicity and increased ROS production [61]. Giorgetti et al. [26] also found a decrease in root length when *A. cepa* seeds were germinated in the presence of 50 nm sized PS-MPs. The particle size of MPs has been considered as an important factor in determining their interaction with tissues and cells, with smaller MPs having a higher chance of being internalized into plant tissues and inducing effects [33,62]. However, experimental setup as well as plant species and age must also be considered when drawing conclusions about MP effects.

Effects of PMMA-MPs on plant growth were far less studied compared to PS-MPs. Dong et al. [39] reported that 100 nm sized PMMA microspheres had no effect on *Brassica campestris* root elongation, which is in good agreement with our results obtained on onion treated with two lower concentrations. Interestingly, at the highest applied PMMA-MP concentration (1 g L^−1^), we recorded a stimulatory effect on the root growth. Although so far, similar findings for PMMA-MPs could not be found in the literature, increased root length was reported upon exposure of different plant species to PS- [34,35,63], PVC- [25], and PET-MPs [64] (reviewed in [65]). Lian et al. [35] correlated increased root growth with enhanced α-amylase activity and consequently a higher amount of soluble sugars required for growth processes, suggesting that MPs can act as “nanocatalysts”.

### 3.3. Effects on Oxidative Stress and Activation of Antioxidant Enzymes

Oxidative stress, which results from the excessive production of ROS molecules, has been proposed as one of the mechanisms of MP-induced phytotoxicity (reviewed in [66]). We found no significant effect on O_2_^•−^ radical formation upon exposure to either MP treatment in the present study. A more prominent effect was recorded on the formation of H_2_O_2_, especially at the highest tested concentration (1 g L^−1^) of MPs of both polymer types. O_2_^•−^ is a relatively short-lived ROS molecule, which is rapidly converted to H_2_O_2_ and O_2_ by the activity of the SOD enzymes. In contrast, H_2_O_2_ is uncharged, more stable, and can pass membranes freely [67]. Lipid peroxidation and protein oxidation, parameters of oxidative stress, were not elevated upon exposure to tested MPs, which indicates that there was no oxidative damage in root cells and corroborates growth results. On the contrary, in other studies performed on *A. cepa* roots, the significantly elevated formation of O_2_^•−^ and hydroxyl radical (·OH) as well as production of MDA were recorded upon exposure to 100 nm sized PS-MPs [61]. Moreover, Giorgetti et al. [26] recorded a significantly increased H_2_O_2_ and MDA content after treatment with 1 g L^−1^ of 50 nm sized PS-MPs. These results confirm the importance of MP size and the stronger phytotoxic effects of smaller MPs compared to the 147 nm MPs used in our research. However, the content of MDA was not significantly changed upon exposure of cucumber roots to 100 nm sized PS-MP [33]. Moreover, Jiang et al. [22] reported that lower concentrations of 100 nm sized PS-MPs (≤50 mg L^−1^) inhibited MDA formation in *V. faba* root cells, while the highest concentration (100 mg L^−1^) significantly increased it. These findings suggest that other factors, including plant species and MP concentration, are also important for MP phytotoxicity.

No significant damage of DNA molecules was recorded in the current study with either PS- or PMMA-MPs, suggesting that MP treatments, tested by Comet assay, do not induce genotoxic effects in onion roots. The Comet test, a simple method for measuring DNA strand breaks in eukaryotic cells, has not yet been employed in testing MP genotoxicity in plant cells, although it was successfully applied to test MP toxic effects in fish larvae [68], earthworms [69], *Drosophila melanogaster* [70], and human colon epithelial cells [71]. As for the cytological parameters, mitotic index was not affected by any of the MP treatments, which is in agreement with no observed changes in root growth. However, we recorded an increased number of cells with chromosomal abnormalities, in particular C-mitosis, which suggests a somewhat negative effect of MPs on the mitotic spindle and chromosome segregation. Other studies performed on onions showed MP-induced cytogenotoxicity through reduced mitotic index and induced chromosomal aberrations and nuclear abnormalities in root cells upon exposure to smaller 50 nm sized PS-MPs [26] and 100 nm sized PS-MPs [61]. In the latter, mitodepressive activity was attributed to the inhibition of cell cycle regulators like CDC2 and the delaying of the cell cycle timing. An increased number of micronuclei was found in *V. faba* roots treated with PS-MPs and PS-NPs, where 100 nm sized PS-MPs induced higher genotoxic damage than larger 5 μm sized PS-MPs [22].

The antioxidant machinery of onion root cells was found to be elevated upon exposure to both types of MPs, especially for enzymes which efficiently scavenge H_2_O_2_, such as CAT and peroxidases. These findings explain the rather low increase in the contents of O_2_^•−^ and H_2_O_2_ and the absence of oxidative damage, as the synchronized function of antioxidant enzymes helps to prevent the ROS-induced damage of cellular biomolecules [72]. On the other hand, ROS act as signal molecules to induce the activation of defense responses. Elevated activities of SOD, PPX, and CAT were also found in roots of *V. faba* upon exposure to PS-MPs of 100 nm size [22] and rice treated with 20 nm sized PS microspheres, indicating that antioxidant enzymes can have a significant role in defense against MP toxicity [58].

### 3.4. Overall Effects—Comparison of PS-MPS and PMMA-MPs

Results obtained with Py-GC-MS suggest that onion roots more readily take up polystyrene-based MPs compared to methyl-methacrylate-based ones, since styrene monomer was recorded in all treatments with PS-MPs, while methyl methacrylate monomer was found only after exposure to the highest tested concentration (1 g L^−1^).

This is in agreement with the overall effects of PS-MPs, which exhibited somewhat more toxic effects in *A. cepa* roots than PMMA-MPs, which could be correlated with more prominent root uptake of PS-MPs. Regarding the root growth, the only significant difference between the PS-MPs and PMMA-MPs was a stimulatory effect of PMMA-MPs at the highest concentration. Pignatelli et al. [73] investigated PE-, PP-, and PVC-MP effects on the seed germination of *L. sativum* and found that PVC-MPs were the most toxic and PP-MPs the least toxic, while in the study of Shi et al. [74], it was found that PE-MPs were more toxic to tomato seedling growth compared to PS- and PP-MPs. Upon exposure of *C. pepo* plants to PP-, PE-, PVC-, and PET-MPs, all MPs exhibited impaired root and shoot growth, although PVC-MPs were found to be the most toxic and PE-MPs the least toxic among all tested microplastics [75].

As for the biochemical parameters, it can be observed that exposure to PS-MPs resulted in the increased formation of ROS molecules and increased contents of MDA and protein carbonyls compared to corresponding PMMA-MP treatments, which was particularly pronounced at the highest tested concentration. These results could be correlated with the lower uptake of PMMA-based MPs. However, the activities of antioxidant enzymes were similarly elevated upon exposure to both types of MPs compared to the control. From the obtained results, we can conclude that even though *A. cepa* roots do not take up PMMA-MPs as readily as PS-MPs, PMMA-based microparticles still induce a response in cell antioxidant machinery. The differential response of biochemical parameters upon exposure of certain plant species to MPs of various polymer composition was recorded in studies by other authors. When the effects of PS-, PE-, and PP-MPs on tomato seedlings were studied, PP-MPs induced a weaker response on the activity of antioxidant enzymes than PS- and PE-MPs [74]. Moreover, PVC-MPs induced a more severe impact on biochemical parameters in *L. sativum* in comparison to PP- and PE-MPs, and authors concluded that garden cress is not able to counteract the toxicity induced by PVC-MPs, and in a minor way, it is not able to counteract PE-MPs over a long exposure time [73]. These results indicate that MPs of different polymer composition may result in varying effects on plant growth and physiology in different plant species.

## 4. Materials and Methods

### 4.1. Characterization and Stability Evaluation of MPs

In this study, we tested the effects of commercial monodisperse polystyrene microparticles (PS-MPs; mean diameter 0.147 ± 0.007 µm) and monodisperse polymethyl methacrylate microparticles (PMMA-MPs; mean diameter 0.105 ± 0.005 µm), purchased from Microparticles GmbH (Berlin, Germany). Both types of MPs were obtained as 5% *w/v* aqueous suspensions.

Microparticle imaging was performed by placing 100 mesh Formvar^®^/Carbon copper grid on a 5 µL droplet of PS or PMMA stock solution for 5 min, after which excess solution was removed and viewed with a monochromatic TF20 (FEI Tecnai G2, FEI, Hillsboro, OR, USA) transmission electron microscope (TEM) containing a Schottky cathode operating at 200 kV. Three replicates (*n* = 3) were investigated for each stock solution.

The stability of 0.01, 0.1, and 1 g L^−1^ solutions of PS-MPs and PMMA-MPs in ultrapure water (exposure medium for root treatment) was determined by measurements of the hydrodynamic diameter (d_H_) using the Dynamic Light Scattering (DLS) method on NanoBrook 90Plus (Brookhaven Instruments, Holtsville, NY, USA) each day during the 3-day period (duration of the root treatment). Results are reported as an average value of 10 measurements, and the size distributions are reported as volume distributions.

### 4.2. Plant Material and Treatment

Onion (*Allium cepa* L.) bulbs were purchased from Sjemenarna d.o.o (Zagreb, Croatia). Prior to the experiment, bulbs were scrapped to remove yellowish-brown scales and their bottom plates in order to expose the apices of the root primordials. Prepared bulbs were placed on the top of the test tubes (one bulb per each test tube), which were filled with ultrapure water (ion-free Milli-Q water, Millipore, 18.2 MΩ-cm resistivity) and placed in the dark for two days. Bulbs with the same initial root length (around 2 cm) were thoroughly washed in ultrapure water and subsequently subjected to exposure with aqueous solutions of PS-MPs and PMMA-MPs, which were prepared by dispersing the MP stock solutions in ultrapure water in order to obtain 0.01, 0.1, and 1 g L^−1^ concentrations, which were determined by our preliminary experiments and data obtained from the literature [22,26,39,43]. The treatments lasted for 72 h at 25 °C. Roots of control bulbs were immersed in ultrapure water for the same period of time under the same conditions. After 72 h, onion bulbs were taken out and the roots were thoroughly rinsed with ultrapure water. For these experiments, 6 onion bulbs were taken for each exposure treatment, including control, and analyzed separately. Exposure experiment was conducted two times.

### 4.3. Root Growth, Mitotic Index and Chromosomal Aberrations

The length of the three longest roots was measured from each control and each treated bulb (6 bulbs per treatment) using a ruler, after which the average length was calculated for each treatment.

Mitotic index (MI) analysis was performed as previously described by Cvjetko et al. [42]). Five bulbs were used for each concentration of PS-MPs and PMMA-MPs and three roots per bulb were examined upon 72 h exposure. Fixed roots were kept in 3:1 ethanol: acetic acid mixture for 24 h or longer, prior to microscope analysis. The roots were stained in 2% acetoorcein at 60 °C for about 10 min. The stained root tips (1–2 mm) were then placed in a drop of 45% acetic acid on a microscopic slide and squashed. Slides were examined using the AXIO Lab A1 ZISS light microscope at 1000× magnification. The MI was calculated as the ratio between the number of mitotic cells and the total number of cells scored (3000) and expressed as percentage. Different types of chromosomal abnormalities were scored: c-mitosis, laggards, anaphase bridges, micronucleus, and stickiness. Percentages of aberrations were calculated as ratio between the total numbers of aberrant cells divided by the total number of dividing cells.

### 4.4. MP Uptake Measurements

Upon harvest, roots were washed with ultrapure water to remove MPs possibly adhered to the tissue, after which they were dried and frozen at −80 °C and lyophilized for 24 h at −64 °C and 0.025 mbar.

To detect and quantify each type of MP in root samples, pyrolysis gas chromatography/mass spectrometry (Py-GC/MS) technique was applied. This technique combines the process of pyrolysis, which is the degradation of high molecular polymer into smaller organic compounds by heating in a pyrolysis chamber. The pyrolysis temperature was set at 600 °C. For each test, 0.3 mg of dried biological material was placed into the deactivated stainless steel sample cup separated by a metal capillary separation column. For analyses of PS-MP and PMMA-MP standards, 2 µL of each commercial monodisperse microparticle solution (Section 4.1) was placed in the sample cup.

Py-GC/MS measurements were carried out using a micro-furnace pyrolyzer (EGA/Py-3030D, Frontier Laboratories Europe, Essen, Germany) equipped with an auto-shot sampler (AS-1020E, Frontier Laboratories). The pyrolyzer was interfaced directly to the split/splitless injection port of a GC/MS instrument (GCMS Shimadzu QP2010 Plus). The GC injection port was connected to a quadrupole mass detector through a separation column (Ultra ALLOY+-5, 30 m × 0.25 mm i.d., coated with 0.5 μm film thickness of 5% diphenyl 95% dimethylpolysiloxane, Frontier Laboratories, Ltd.) and a vent-free GC/MS adapter (Frontier Laboratories, Ltd.). The detailed analytical conditions are listed in Table 3. The qualifications and identifications of peaks in the chromatograms were confirmed by comparing the mass spectrum of each peak in the pyrogram with those in data search libraries of F Search all in one (ver.3.6) (Frontier Laboratories Ltd., Japan).

### 4.5. Protein Extraction

Protein extraction was performed by grinding 0.1 g of fresh root tissue in 1.5 mL of 100 mM potassium phosphate buffer, pH 7.0, using a pre-cooled mortar and pestle. A total of 50 mg of insoluble polyvinylpyrrolidone (PVP) was added to the tissue prior to grinding. Obtained homogenates were centrifuged for 15 min at 20,000× *g* and 4 °C, after which the supernatants were transferred to clean tubes and recentrifuged for 60 min under the same conditions. Protein concentration in the samples was determined according to the Bradford method [76], with bovine serum albumin (BSA) as a standard. These samples were used for measurement of total ROS content, protein carbonyls, and activities of antioxidant enzymes.

### 4.6. ROS Content

Contents of superoxide radical (O_2_^•−^) and hydrogen peroxide (H_2_O_2_) were determined by employing fluorescent probes dihydroethidium (DHE) and 2′,7′-dichlorodihydrofluorescein diacetate (H_2_DCF-DA), respectively. Prior to each measurement, 50 μL of 20 μM DHE or H_2_DCF-DA was added to the same volume (50 μL) of the protein extract (Section 4.5) in a 96-well plate. Fluorescence was read using microplate reader (GloMax^®^-Multi Detection System Promega, Madison, WI, USA) after short agitation. For DHE, excitation wavelength was set at 525 nm and emission between 580 and 640 nm, and for H_2_DCF-DA, excitation was set at 490 nm and emission between 510 and 570 nm. All results are expressed as percentage of control (non-treated sample).

### 4.7. MDA and Protein Carbonyl Content

To assess the damage level of lipid molecules, we implemented the modified method of Heath and Packer [77]. A total of 0.1 g of fresh tissue was extracted in 1.3 mL of a reaction mixture consisting of 0.3% (*w/v*) thiobarbituric acid (TBA) and 10% (*w/v*) TCA, using a mortar and pestle. Homogenates were incubated for 30 min at 95 °C, after which they were cooled in an ice bath and centrifuged for 15 min at 20,000× *g* and 4 °C. The content of malondialdehyde (MDA) in obtained supernatants was measured at the wavelength of 532 nm with the subtraction of the value measured at the wavelength of 600 nm as a correction for nonspecific turbidity. The MDA content was calculated using the MDA molar absorption coefficient of 155 mM^−1^ cm^−1^. Results are expressed as μmol mg^−1^ of dry weight.

For the protein carbonyl content measurement, the method of Levine et al. [78] was applied. A total of 200 μL of protein extracts (Section 4.5) was mixed with 300 μL of 10 mM 2,4-dinitrophenylhydrazine (DNPH) dissolved in 2 M HCl and incubated at room temperature for 1 h in the dark. A total of 500 μL of 10% (*w/v*) TCA was combined with each sample for protein precipitation. Samples were incubated for 10 min at −20 °C and subsequently centrifuged for 10 min at 20,000× *g* and 4 °C. Supernatants were discarded and pellets were thoroughly washed 3× with 500 μL solution of ethanol/ethylacetate (1:1, *v/v*) to remove excess reagent. Pellets were then resuspended in 1 mL of 6 M urea dissolved in 20 mM potassium phosphate buffer (pH 2.4). Absorbance was read at the wavelength of 370 nm against the reference prepared for each sample in a reaction mixture devoid of DNPH. Absorbance of each sample was also measured at the wavelength of 280 nm in order to estimate protein recovery. Protein carbonyl concentration was calculated using the aliphatic hydrazones’ molar absorption coefficient of 22 mM^−1^ cm^−1^. Results are expressed as μmol mg^−1^ of proteins.

### 4.8. Comet Assay

To estimate the DNA damage level with Comet assay, we implemented the modified method by Cvjetko et al. [42,79]. Nuclei were mechanically isolated from root tissue in 400 mM Tris-HCl (pH 7.5) at 4 °C, after which 50 μL of nuclei suspension was combined with 50 μL of 1% (*w/v*) low melting agarose and placed onto a slide. After 10 min denaturation (for DNA unwinding) and 20 min of electrophoresis (26 V and 300 mA) in a fresh buffer (1 mM Na_2_EDTA and 300 mM NaOH, pH 13), slides were neutralized with 400 mM Tris-HCl (pH 7.5), air-dried, and stained with 70 μL of GelStarTM Nucleic Acid Gel Stain (Lonza, Rockland, ME, USA). Image processing program Image J was employed for the image analysis using OpenComet v1.3.1 plugin [80] to assess the tail DNA percentage (%tDNA), which is a primary measure of DNA damage.

### 4.9. Activities of Antioxidant Enzymes

Activity measurements of all antioxidant enzymes were conducted using the UV/visible spectrometer (ATI UNICAM UV4, Cambridge, UK) at 25 °C.

Activity of SOD (EC 1.15.1.1) was assayed by employing the method of Beauchamp and Fridovich [81], which is based on the nitroblue tetrazolium (NBT) reduction inhibition. For determination of the maximum absorbance, the reaction mixture (13 mM methionine, 75 μM NBT, 0.1 M EDTA, and 2 mM riboflavin) was incubated for 8 min in a 15 W light box, and formazan produced by NBT photo reduction was measured at the wavelength of 560 nm. The certain volume of protein extract (Section 4.5), which is required to inhibit the NBT reduction rate for 50% (one unit of SOD activity), was then added to the reaction mixture. The measurement was conducted as already explained. Specific activity of SOD was expressed as U of SOD activity mg^−1^ of proteins.

For APX (EC 1.11.1.11) activity measurement, the method published by Nakano and Asada [82] was implemented. A total of 180 μL of the protein extract (Section 4.5) was combined with 800 μL of reaction mixture (50 mM potassium phosphate buffer, pH 7.0, and 10 mM EDTA), 10 μL of 0.1 mM ascorbic acid, and 10 μL of 12 mM H_2_O_2_. The decrease in absorbance, caused by the consumption of ascorbate (ε = 2.8 mM^−1^ cm^−1^), was measured at the wavelength of 290 nm. APX specific activity was indicated as μmol of oxidized ascorbate min^−1^ mg^−1^ proteins.

PPX (EC 1.11.1.7) activity was also assayed according to Nakano and Asada [82]. A total of 20 μL of the protein extract (Section 4.5) was added to 980 μL of the reaction mixture consisting of 50 mM potassium phosphate buffer, pH 7.0, 20 mM pyrogallol, and 1 mM H_2_O_2_. The increase in absorbance, caused by the pyrogallol (ε = 2.6 mM^−1^ cm^−1^) oxidation, was measured at the wavelength of 430 nm. PPX specific activity was calculated as μmol of purpurogallin min^−1^ mg^−1^ proteins.

Measurement of CAT (EC 1.11.1.6) activity was performed according to the method published by Aebi [83]. A total of 30 μL of the protein extract (Section 4.5) was combined with 970 μL of the reaction mixture containing 50 mM potassium phosphate buffer (pH 7.0) and 10 mM H_2_O_2_. The decrease in absorbance, which indicates the decomposition of H_2_O_2_ (ε = 36 mM^−1^ cm^−1^), was recorded at the wavelength of 240 nm. CAT specific activity was calculated as μmol of decomposed H_2_O_2_ min^−1^ mg^−1^ proteins.

### 4.10. Statistical Analysis

Data acquired in this research were analyzed with one-way ANOVA and Duncan post hoc test in STATISTICA 12.0 (Stat Soft Inc, Palo Alto, CA, USA). Each data point represents a mean value ± standard error (SE) of 12 replicates obtained in two independent experiments. Differences between means were considered statistically significant at *p* ≤ 0.05.

## 5. Conclusions

In this study, we analyzed the effects of microplastics composed of two different polymers, the frequently studied PS-MPs, one of the leading pollutants found in both aquatic and terrestrial ecosystems, and PMMA-MPs, commonly used in cosmetics production, but seldom investigated for phytotoxic effects, on *A. cepa* roots. Results obtained with Py-GC-MS suggest that both types of MPs are taken up by roots cells, although the uptake of PS-MPs was more efficient compared to PMMA-MPs. Neither MP induced any (cyto)toxic effect on root growth. The strongest response to both types of MPs was the activation of antioxidant enzyme machinery (SOD and especially CAT and peroxidases) of root cells, which consequently successfully decomposed ROS and prevented the oxidative damage. PMMA-MPs in general had a stimulating effect on root growth, which together with the strong activation of antioxidant enzymes, suggests their nanocatalytic activity.

## Figures and Tables

**Table 1:** Temporal analysis of changes in hydrodynamic diameter (d_H_) of 0.01, 0.1, and 1 g L^−1^ PS-MPs and PMMA-MPs in ultrapure water.

	PS-MPs (g L^−1^)			PMMA-MPs (g L^−1^)		
	0.01	0.1	1	0.01	0.1	1
0 min	141.1 ± 23.8 ^b^	144.7 ± 1.6 ^b^	142.4 ± 1.3 ^a^	111.0 ± 1.4 ^b^	114.0 ± 0.8 ^a^	112.0 ± 0.7 ^a^
24 h	160.7 ± 1.7 ^a^	152.0 ± 1.6 ^a^	139.3 ± 0.5 ^ab^	111.0 ± 1.5 ^b^	110.0 ± 0.9 ^b^	97.5 ± 0.8 ^c^
48 h	157.7 ± 1.5 ^a^	136.1 ± 2.3 ^c^	136.9 ± 1.4 ^b^	117.2 ± 0.7 ^a^	105.2 ± 1.3 ^c^	99.0 ± 0.8 ^c^
72 h	159.3 ± 2.2 ^a^	131.9 ± 1.6 ^d^	131.7 ± 1.6 ^c^	101.3 ± 2.3 ^c^	113.9 ± 1.3 ^a^	103.2 ± 1.0 ^b^

Values are means of 10 measurements ± standard deviations. Changes in d_H_ of each concentration and MP type were analyzed separately. If values in each column are marked with different letters, temporal changes in d_H_ are significantly different at *p* ≤ 0.05 (a one-way ANOVA followed by Duncan’s post hoc test).

**Table 2:** Mitotic index and chromosome aberrations in control *A. cepa* root meristem cells and after 72 h treatment with 0.01, 0.1, and 1 g L^−1^ of PS-MPs and PMMA-MPs.

MP Treatment	Conc. (g L^−1^)	No. Dividing Cells	Mitotic Index	% Aberrant Cells	No. C-mitosis	No. Multiple Anaphase	No. Laggards	No. Anaphase Bridges	No. Sticky Chromosomes	No. Micronucleus
Control	0	222	7.4 ± 0.2 ^a,A^	1.3 ± 0.9 ^c,C^	2 ^d,C^	0 ^a,A^	1 ^a,A^	0 ^a,A^	0 ^a,A^	0 ^a,A^
PS	0.01	236	7.9 ± 0.3 ^a^	6.1 ± 1.0 ^b^	10 ^b^	0 ^a^	1 ^a^	0 ^a^	2 ^a^	0 ^a^
	0.1	276	9.2 ± 0.9 ^a^	6.4 ± 1.2 ^b^	11 ^b^	1 ^a^	3 ^a,^*	0 ^a^	0 ^a^	2 ^a^
	1	239	8.0 ± 0.6 ^a^	12.0 ± 1.0 ^a^	22 ^a^	0 ^a^	2 ^a^	1 ^a^	1 ^a^	2 ^a^
PMMA	0.01	213	7.1 ± 0.3 ^A^	7.2 ± 1.5 ^B,^*	14 ^B^	0 ^A^	1 ^A^	0 ^A^	0 ^A^	0 ^A^
	0.1	221	7.4 ± 0.3 ^A^	6.8 ± 1.0 ^B^	15 ^B^	0 ^A^	0 ^A^	0 ^A^	0 ^A^	0 ^A^
	1	210	7.0 ± 0.3 ^A^	12.1 ± 1.7 ^A,^*	25 ^A,^*	0 ^A^	0 ^A^	0 ^A^	0 ^A^	0 ^A^

Values are means ± standard errors of measurements obtained in 5 different roots, each with 600 cells (n = 3000). If values are marked with different letters or asterisk, the treatments are significantly different at *p* ≤ 0.05 (one-way ANOVA followed by Duncan post hoc test); small letters mark the differences among different concentrations of PS-MPs and control, capital letters mark the differences among different concentrations of PMMA-MPs and control, and asterisk (*) denotes difference between the same concentration of different MP types.

**Table 3:** Analytical conditions for Py-GC/MS used to detect MPs in onion root samples.

Instrument	Parameters	Settings
Pyrolyzer	Furnace temperature	600 °C
	Interface temperature	300 °C
GC	Injection port temperature	300 °C
	Column oven temperature	40 °C (2 min hold)–320 °C (20 °C min^−1^, 16 min hold)
	Flow Control mode	Pressure
	GC/MS interface temperature	300 °C
	Injection mode	Split (split ratio: 1:16)
	Carrier gas	Helium (column flow rate: 0.87 mL min^−1^)
MS	Ion source temperature	250 °C
	Ionization method	Electron ionization (EI), 70 eV
	Scan range	*m/z* 29−350

## Data Availability

Not applicable.

## Supplementary Materials

The following supporting information can be downloaded at: https://www.mdpi.com/article/10.3390/plants12040747/s1, Figure S1: Ion chromatogram of styrene monomer (M), dimer (D) and trimer (T) from pyrolysis GC-MS with characteristic *m/z* values. (A) standard sample of PS-MPs; (B) Control (untreated *Allium cepa* roots); *A. cepa* roots treated with (C) 0.01 g L^−1^ PS-MPs; (D) 0.1 g L^−1^ PS-MPs; (E) 1 g L^−1^ PS-MPs.; Figure S2: Ion chromatogram of methyl methacrylate (MM) from pyrolysis GC-MS with characteristic *m/z* values. (A) standard sample of PMMA-MPs; (B) Control (untreated *Allium cepa* roots); *A. cepa* roots treated with (C) 0.01 g L^−1^ PMMA-MPs; (D) 0.1 g L^−1^ PMMA-MPs; and (E) 1 g L^−1^ PMMA-MPs.
