# Supplementary material for: Phytotoxic Effects of Polystyrene and Polymethyl Methacrylate Microplastics on Allium cepa Roots
Source: Plants (Basel). 2023 Feb 7;12(4):747. doi: 10.3390/plants12040747 (PMC9959832; doi:10.3390/plants12040747)
Supplement: Supplementary file 1 [file plants-12-00747-s001.zip › Supplement S1_PS-MPs_whole chromatogram.pdf]

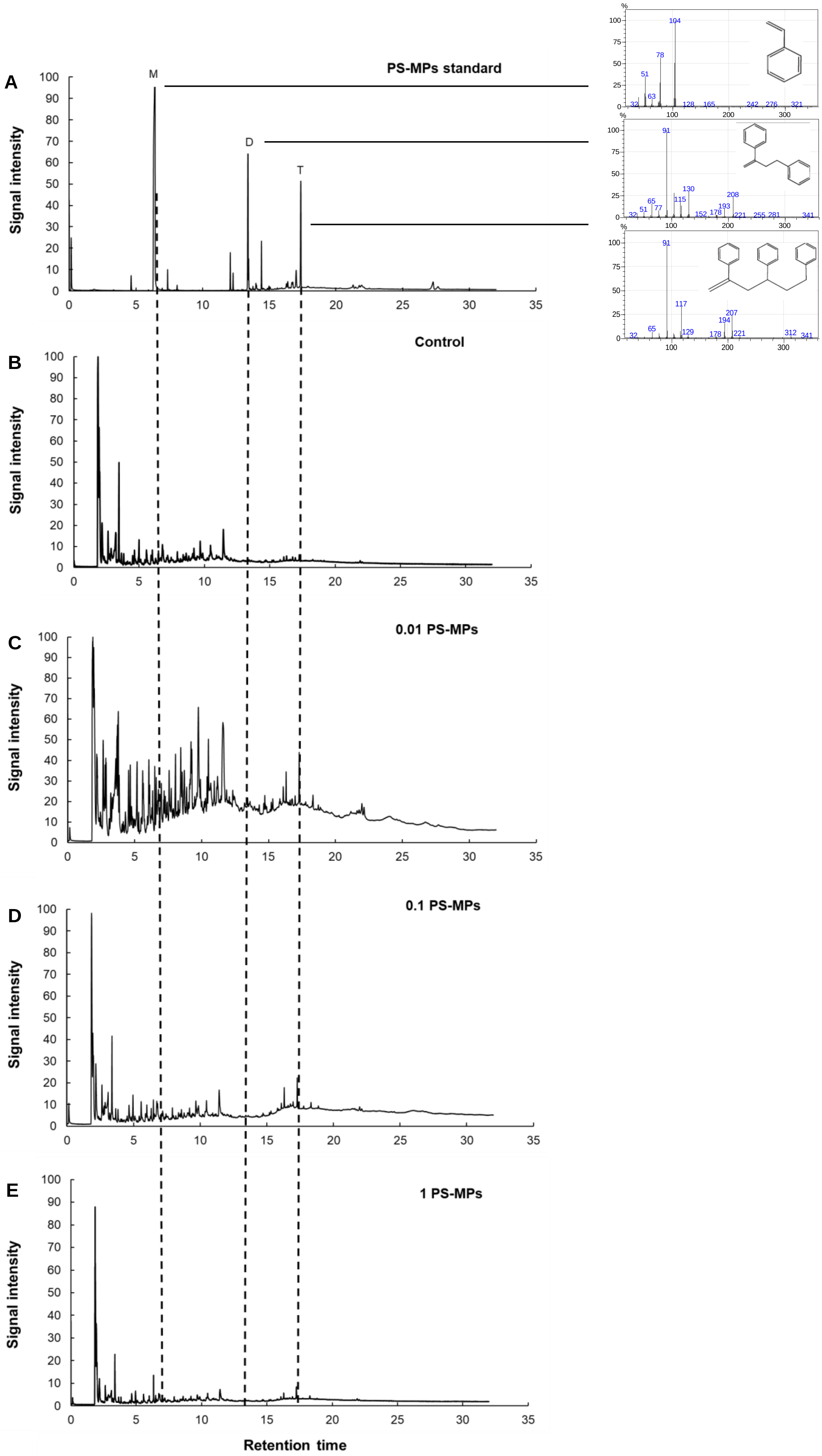

A
PS-MPs standard
M
D
T
B
Control
C
0.01 PS-MPs
D
0.1 PS-MPs
E
1 PS-MPs
Signal intensity
Retention time

Supplemental Figure S1. Ion chromatogram of styrene monomer (M), dimer (D) and trimer (T) from pyrolysis GC-MS with characteristic *m/z* values. A) standard sample of PS-MPs; B) Control (untreated *Allium cepa* roots); *A. cepa* roots treated with C) 0.01 g L^{-1} PS-MPs; D) 0.1 g L^{-1} PS-MPs; E) 1 g L^{-1} PS-MPs.
