# Supplementary material for: Phytotoxic Effects of Polystyrene and Polymethyl Methacrylate Microplastics on Allium cepa Roots
Source: Plants (Basel). 2023 Feb 7;12(4):747. doi: 10.3390/plants12040747 (PMC9959832; doi:10.3390/plants12040747)
Supplement: Supplementary file 1 [file plants-12-00747-s001.zip › Supplement S2_PMMA-MPs_whole chromatogram.pdf]

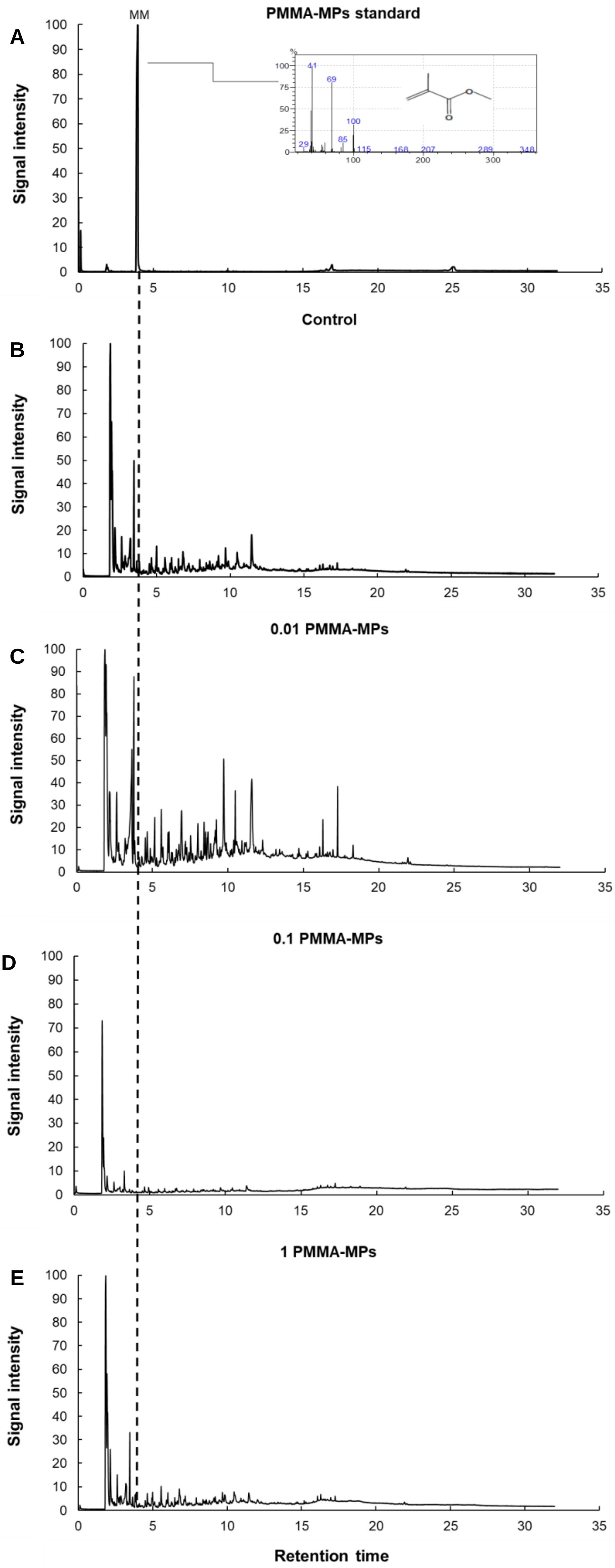

PMMA-MPs standard
A
MM
Signal intensity
41
69
100
85
29
115
168
207
289
348
Control
B
0.01 PMMA-MPs
C
0.1 PMMA-MPs
D
1 PMMA-MPs
E
Retention time

Supplemental Figure S2. Ion chromatogram of methyl methacrylate (MM) from pyrolysis GC-MS with characteristic *m/z* values. A) standard sample of PMMA-MPs; B) Control (untreated *Allium cepa* roots); *A. cepa* roots treated with C) 0.01 g L^{-1} PMMA-MPs; D) 0.1 g L^{-1} PMMA-MPs; and E) 1 g L^{-1} PMMA-MPs.
